# Similar group mean scores, but large individual variations, in patient-relevant outcomes over 2 years in meniscectomized subjects with and without radiographic knee osteoarthritis

**DOI:** 10.1186/1477-7525-2-38

**Published:** 2004-07-27

**Authors:** Przemyslaw T Paradowski, Martin Englund, Ewa M Roos, L Stefan Lohmander

**Affiliations:** 1 Department of Orthopedics, Lund University Hospital, SE-221 85 Lund, Sweden; 2 Department of Orthopedics, Medical University Hospital, Zeromskiego 113, 90-549 Lodz, Poland

**Keywords:** osteoarthritis, knee, meniscus, follow-up, patient-relevant outcome

## Abstract

**Background:**

Epidemiological studies have, so far, identified factors associated with increased risk for incident or progressive OA, such as age, sex, heredity, obesity, and joint injury. There is, however, a paucity of long-term data that provide information on the nature of disease progression on either group or individual levels. Such information is needed for identification of study cohorts and planning of clinical trials. The aim of the study was, thus, to assess the variation in pain and function on group and individual level over 2 years in previously meniscectomized individuals with and without radiographic knee osteoarthritis (OA).

**Methods:**

143 individuals (16% women, mean age at first assessment 50 years [range 27–83]) were assessed twice; approximately 14 and 16 years after isolated meniscectomy, with a median interval of 2.3 years (range 2.3–3.0). Radiographic OA (as assessed at the time of second evaluation) was present in the operated knee in 40%, and an additional 19% had a single osteophyte grade 1 in one or both of the tibiofemoral compartments. Subjects completed the self-administered and disease-specific Knee injury and Osteoarthritis Outcome Score (KOOS).

**Results:**

There were no significant changes in the group mean KOOS subscale scores over the 2-year period. However, a great variability over time was seen within individual subjects. Out of 143 subjects, 16% improved and 12% deteriorated in the subscale Pain, and 13% improved and 14% deteriorated in the subscale ADL ≥ 10 points (the suggested threshold for minimal perceptible clinical change). Similar results were seen for remaining subscales.

**Conclusion:**

Group mean scores for this study cohort enriched in incipient and early-stage knee OA were similar over 2 years, but pain, function and quality of life changed considerably in individuals. These results may be valid also for other at risk groups with knee OA, and motivate further careful examination of the natural history of OA, as well as properties of the OA outcome instruments used. Longitudinal outcome data in OA studies need to be analyzed both on an individual and a group level.

## Background

Drugs that may slow or halt the breakdown of cartilage and other joint tissues in osteoarthritis (OA) and possibly improve symptoms and function are now being developed in the pharmaceutical industry. The potential availability of disease modifying OA drugs has focused attention on our relative lack of information on the 'natural disease history' of OA with regard to changes in symptoms, functional limitations, joint structure and other markers of disease change [1].

Epidemiological studies have identified factors associated with increased risk for incident or progressive OA, such as age, sex, heredity, obesity, and joint injury, pain, alignment, or laxity. There is, however, a paucity of long-term data that document the rate and nature of natural OA disease progression on either group or individual levels. Such information is needed for identification of study cohorts and planning of clinical trials of disease modifying OA drugs. Even more importantly, knowledge of natural disease progression in different patient groups will be needed to select those future groups that may benefit from such drugs.

Only a few of the previously published studies have presented information on longitudinal variation in pain and function in the natural history of knee OA. The "Bristol 500 OA study" noted, that although pain changed little on a group level over a 3-year follow-up period, it varied greatly in individuals, with some subjects reporting marked improvements. Similarly, a minority improved functionally [2-4].

Yet another report suggested that most patients with OA attending rheumatology clinics do not deteriorate radiographically or symptomatically over an 11-year period [5]. A more recent report stated that 42–44% of community-recruited knee OA individuals did not change in physical functioning over a 3-year study [6]. Most investigations of the natural history of OA have been concerned with radiographic rather than clinical changes. For example, it was reported that the radiographic Kellgren and Lawrence classification score of 1 could represent incipient OA and be predictive of later development of more advanced radiographic features of OA [7]. MRI may be more responsive to change in early-stage OA than plain radiography [8].

However, outcome is usually heterogeneous: study subjects may report improvement or deterioration while they do not change radiographically over the time period assessed. It may also be that a few individuals alone generate much of any change detected at group level [9-11]. A further confounding factor in the longitudinal assessment of OA is the potential influence of the population from which the study group was recruited; a study group recruited from e.g. a specialist outpatient clinic is likely to have, on the average, more severe disease and may be at different risk to progress over time than a study group recruited from the community.

The objective of this investigation was to assess both group and individual variation in knee pain, function and quality of life over two years in a study group enriched in incipient and early-stage radiographic knee OA.

## Methods

### Patients

**Figure 1:**
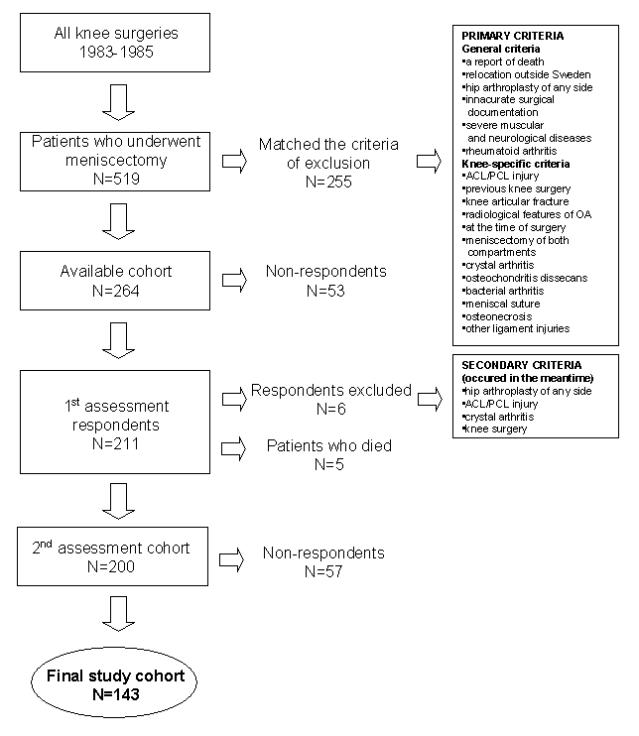
**Flow chart presenting the inclusion and exclusion criteria for patients. **ACL = anterior cruciate ligament, PCL = posterior cruciate ligament, OA = osteoarthritis.

Approval was obtained from the Research Ethics Committee of the Medical Faculty of Lund University, Sweden. All patients who underwent meniscectomy between 1983 and 1985 were identified by searching the surgical records at the Department of Orthopedics, Lund University Hospital. In this period 552 meniscectomies were performed. Inclusion and exclusion criteria (Figure 1) were used to identify 264 former patients who, in 1998, were sent a self-administered questionnaire evaluating their knee-specific symptoms and knee function.

Out of 211 individuals (80%) who returned the questionnaires, 6 were excluded because they matched one of the exclusion criteria. At 2 years after the first assessment 5 subjects had died, but the remaining 200 individuals were asked to provide a second evaluation using an identical questionnaire. Replies were received from 143 (72%). Of these 143 participants, 102 were meniscectomized by open surgery, and 41 by arthroscopy. Nineteen underwent an additional meniscus operation in the index knee.

All re-operations were performed within 3 years after the original meniscectomy. Twenty-three participants were treated with subsequent meniscectomy of the contralateral knee. One of them underwent high tibial osteotomy and 1, because of OA, received a knee prosthesis in the contralateral knee. Data concerning subsequent surgeries were based on the medical records of Lund University Hospital and on self-reported information.

### Radiographic assessment

At the time of the participants' second evaluation with questionnaires, standing anteroposterior (AP) radiographs of both knees were taken in 15 degrees of flexion using a CGR Phasix 60 generator at 70 kV, 16 mA, film-focus distance 1.5 m (CGR, Liège, Belgium). Ten out of the 143 participants (7%) declined the radiographic examination. All AP radiographs of the tibiofemoral joints from the follow-up were assessed for joint space narrowing (JSN) and osteophytes according to the atlas from Osteoarthritis Research Society International (OARSI) [12].

The presence of these features was graded on a 4-point scale (range 0–3, with 0 = no evidence of bony changes or JSN). We considered radiographic knee OA to be present if any of the following criteria was achieved in any of the 2 tibiofemoral compartments: JSN ≥ grade 2 or the sum of the 2 marginal osteophyte grades from the same compartment ≥ 2, or JSN grade 1 in combination with an osteophyte grade 1 in the same compartment [13,14]. This cut-off approximates grade 2 knee OA or worse based on the Kellgren and Lawrence scale [15].

### Disease-specific questionnaire

The Knee injury and Osteoarthritis Outcome Score (KOOS, Swedish version LK 1.0) is a 42-item self-administered knee-specific questionnaire based on the WOMAC Osteoarthritis Index [16,17]. KOOS was developed to be used for short- and long-term follow-up studies of knee injuries, and it comprises 5 subscales: Pain, Symptoms, Activities of Daily Living (ADL), Sports and Recreation Function (Sport/Rec) and knee-related Quality of Life (QOL). A separate score ranging from 0 to100, where 100 represents the best result, is calculated for each subscale.

The questionnaire and scoring manual can be downloaded from . The KOOS is valid, reliable and responsive in follow-up of meniscectomy [17], anterior cruciate ligament reconstruction [18] and total knee replacement for OA [19]. The participants completed the KOOS questionnaire answering questions on their operated index knee.

### Change

The minimal perceptible clinical improvement (MPCI) represents the difference on the measurement scale associated with the smallest change in the health status detectable by the individual. Since the KOOS questionnaire contains the full and original version of the WOMAC LK 3.0 index, we used the MPCI as described for WOMAC [20]. Thus, a level of 10 points or more of improvement or decline was operationally used as a cut-off representing a clinically perceptible difference. The sensitivity of the questionnaire has been established [21].

### Data collection and statistics

If questions were left unanswered in any part of the questionnaire, we returned the questionnaire to be completed. The questionnaires were then completed fully. The Mann-Whitney U-test was used to determine differences between the groups. *P*-values for categoric data were calculated with Fisher's exact test. All tests were 2-tailed and a *P*-value of ≤ 0.05 was considered statistically significant (SigmaStat, version 2.0, for Windows).

## Results

### Group level

The study group comprised 143 individuals, of whom 23 (16%) were women. The participants' mean age at the first follow-up was 51 (range 27–83) years. The assessment was carried out twice: at approximately 14 and 16 years after the surgery, with a median interval of 2.3 (range 2.3 to 3.0) years. Fifty-three (40%) of the 133 individuals who had undergone radiographic examination had radiographic tibiofemoral OA in their index (operated) knee (21% women, age range 29–83, mean 53) and 80 were classified as non having OA (11% women, age range 27–82, mean 50). An additional 25 (19%) (not classified as radiographic OA) had a single osteophyte grade 1 in either one or both tibiofemoral compartments.

**Figure 2:**
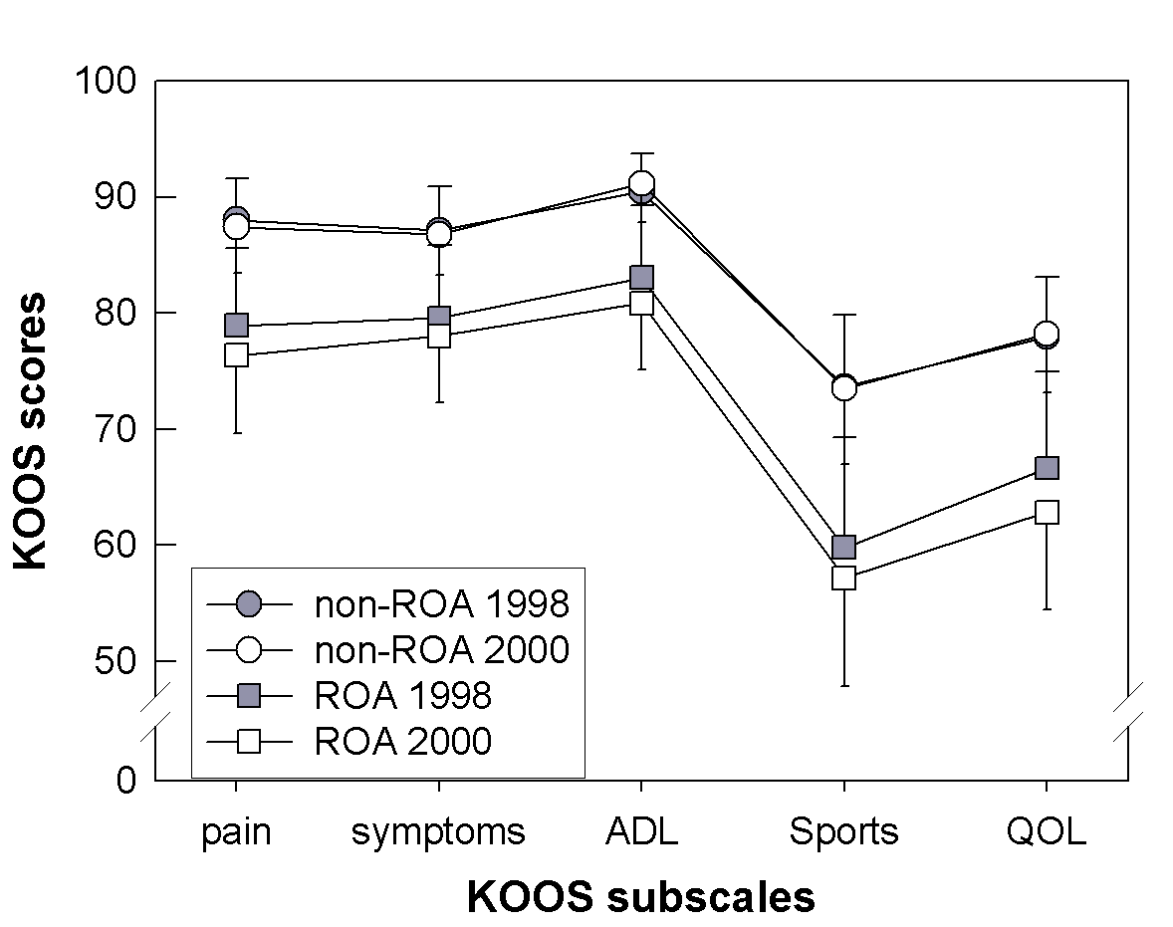
**Group mean KOOS scores for patients assessed in 1998 and 2000. **Group mean KOOS scores for patients with (n = 53) and without (n = 80) radiographic osteoarthritis (ROA) assessed in 1998 and 2000. Possible score range 0 to 100, with 100 representing the best result. ADL – Activities of Daily Living, QOL – knee-related Quality of Life. Bars present ± 95% confidence intervals. The bars going upwards have wider caps. Note vertical axis break.

Mean scores for the KOOS subscales at the first assessment did not change significantly over the 2-year study period (Table 1). Moreover, there were no significant changes in group mean subscale scores over 2 years when participants were divided into those with or without radiographic OA in the index knee (Table 1, Figure 2). However, individuals with radiographic OA scored worse at both examinations than did those without radiographic OA. The differences between those with and without OA were statistically significant for KOOS Pain Δ = 11 points (*P *= 0.004), other Symptoms Δ = 9 points (*P *= 0.013), ADL Δ = 10 points (*P *= 0.003), Sport/Rec Δ = 17 points (*P *= 0.005), and QOL Δ = 16 points (*P *= 0.003) assessed in 2000, and in the dimensions Sport/Rec Δ = 14 points (*P *= 0.020) and QOL Δ = 12 points (*P *= 0.041) evaluated in 1998.

**Table 1:** KOOS scores overall and in patients without and with radiological signs of OA

KOOS subscales		Patients						p-values
			
		Total group		non-ROA		ROA		non-ROA vs. ROA
		n = 143		n = 80		n = 53		
		1998	2000	1998	2000	1998	2000	2000
pain	mean	85	84	88	87	79	76	0.008
	median	94	94	94	94	86	83	
	SD	20	21	16	18	24	25	
	range	19–100	25–100	39–100	25–100	19–100	25–100	
symptoms	mean	85	84	87	87	80	78	0.013
	median	93	89	93	93	89	82	
	SD	19	18	17	16	23	21	
	range	14–100	14–100	25–100	18–100	14–100	14–100	
ADL	mean	88	88	90	91	83	81	0.004
	median	99	97	99	99	94	90	
	SD	18	18	15	15	23	21	
	range	18–100	31–100	44–100	34–100	18–100	31–100	
sports/rec	mean	69	68	74	76	60	57	0.007
	median	80	80	80	85	60	60	
	SD	31	32	28	28	34	34	
	range	0–100	0–100	0–100	0–100	0–100	0–100	
QOL	mean	75	73	78	78	67	63	0.005
	median	81	81	81	84	69	63	
SD	26	27	23	23	30	30		
range	0–100	6–100	25–100	6–100	0–100	13–100		

Mean, median, standard deviation and range of KOOS scores overall and in patients without and with radiological signs of OA. Note that 10 patients out of 143 did not undergo radiographic examination. *P*-values for comparison between KOOS subscale results in patients with and without OA in year 2000 are presented.

We analyzed separately those subjects (N = 57) that did not participate in the second assessment. Their mean KOOS scores at the first examination did not differ significantly from the remainder of the study cohort, indicating little or no inclusion bias for the second follow-up (data not shown). The scores in the 5 patients that underwent additional surgery (e.g. osteotomy, knee arthroplasty) did not differ significantly from the rest of the group.

### Individual study subject changes

In spite of the lack of change on a group level, we found substantial intra-individual variability in the questionnaire subscale scores measured 2 years apart. Out of the total 143 study subjects, 40 had either improved or deteriorated (n = 23 (16%) and n = 17 (12%), respectively) 10 points or more for the KOOS subscale Pain. Of the 23 subjects who had improved in their pain score by these criteria, 14 had also improved in the subscale Symptoms, 17 in ADL, 16 in Sports/Rec, and 17 in QOL.

Only 1 of these subjects deteriorated in Symptoms, and 2 in Sports/Rec, none in the other subscales. Of the 17 subjects who deteriorated in Pain, 13 similarly deteriorated in Symptoms, 12 in ADL, 10 in Sports/Rec, and 10 in QOL. When evaluating those who had undergone radiographic examination, there were no significant differences in variability detected whether the subject had radiographic tibiofemoral OA or not (*P *= 0.24, Table 2).

**Table 2:** The percentage of patients improving, not changing, or deteriorating for KOOS subscales over time

			non-ROA			ROA	
KOOS subscales	cut-off		n = 80			n = 53	
		+	no change	--	+	no change	--
			%			%	
pain	10	13	76	11	21	66	13
	20	6	88	6	8	87	6
symptoms	10	16	69	15	26	55	19
	20	6	86	8	13	77	9
ADL	10	9	79	13	19	64	17
	20	5	86	9	15	79	6
sports/rec	10	19	60	21	28	42	30
	20	11	76	13	21	64	15
QOL	10	20	56	24	26	57	17
20	5	88	8	15	75	9	

The percentage of patients, with and without radiographic osteoarthritis (ROA), improving, not changing, or deteriorating for KOOS subscales over the 2 year study period. For definition of ROA see methods. Two cut-offs for change (≥ 10 and ≥ 20 points) are presented.

We also evaluated a stricter cut-off of 20 points or more as used for the OARSI responder criteria, as opposed to minimal clinically perceptible change [22]. With this cut-off, in total 19 patients fulfilled the criterion for improvement or deterioration (n = 9 (6%), n = 10 (7%), respectively) in KOOS Pain. Among the subjects with radiographic OA, 3 (6%) improved and 4 (7%) deteriorated by 20 points or more. Corresponding numbers for those without radiographic OA were 5 (6%) for both improvement and deterioration.

**Figure 3:**
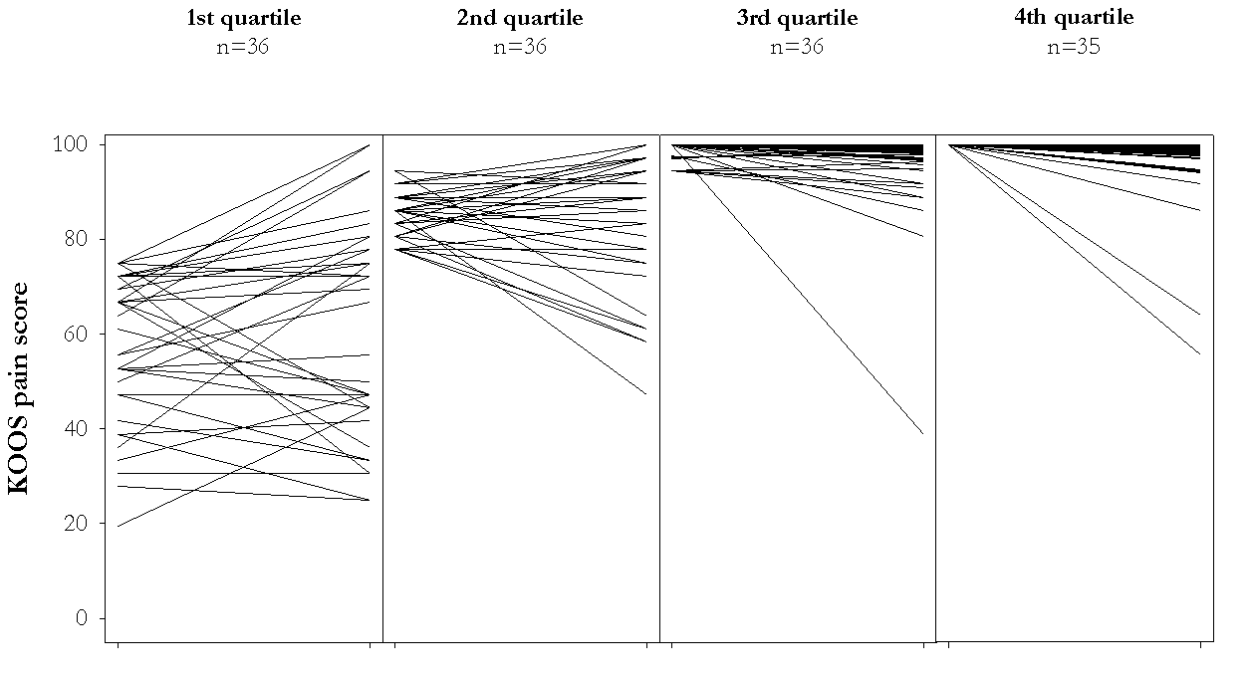
**KOOS Pain subscale. **Patients are divided into 4 subgroups (quartiles) according to the score at entry. Each line represents one patient visualizing the score in 1998 (left endpoint of line) and in 2000 (right endpoint of the same line).

In order to explore these changes in more detail, the subjects were divided into quartiles, according to KOOS Pain score at the first assessment (Figure 3). The most noticeable changes were found in the quartile representing the worst scores: 21 of 36 (58%) subjects showed a change of 10 points or more in either direction. A corresponding change was seen in 11 (31%) individuals from the second worst quartile and in only 9 (25%) from the second best and best quartiles (6 and 3 subjects, respectively). Comparable results were seen for the other subscales of KOOS (data not shown).

## Discussion

We found no significant change over 2 years in the average patient-relevant outcome scores for this study group of individuals who had undergone meniscectomy about 15 years earlier, even though the group was highly enriched in early-stage and incipient radiographic knee OA. However, we found substantial change in the self-report for individual subjects over the same time period.

The generally worse KOOS scores for the individuals with radiographic knee OA, compared to those without, are consistent with earlier reports. Thus, the Baltimore Longitudinal Study of Aging reported that patients with a Kellgren-Lawrence score of 1 were almost twice as likely to report ever having knee joint pain compared with those who had a score of zero. The strength of the association increased with increasing Kellgren and Lawrence score [23].

Similarly, there was in meniscectomized individuals evidence for a graded increase in pain and functional limitations with increasing severity of radiographic signs of OA [24]. However, a discrepancy between knee pain and radiographic features of knee OA has also been noted, both cross-sectionally and longitudinally [3,24,25]. Depression and lack of muscle strength have been shown to better explain pain than radiographic findings [26-28].

### Individual vs. group analysis

Few reports have explored OA symptom variation on an individual level [2-4]. A detailed comparison of our results with earlier reports is difficult, since they were conducted before validated and patient-relevant OA disease-specific measurement tools had been widely introduced. The "Bristol OA 500" were patients with advanced radiographic knee OA and a mean age of 65 years recruited from a hospital based rheumatology clinic.

In contrast, the mean age of the present study cohort was 50 years, with 2/5 having mild-moderate radiographic OA, and another 1/5 incipient radiographic changes. Further, the cohort reported on here was recruited from a group of individuals that had undergone isolated meniscectomy 15 years earlier, but independent of their subsequent symptom level or disease history. The mean scores of our study group were relatively good and not representative of subjects with advanced OA seeking medical care.

The rationale for investigating this particular cohort at this time after surgery was its enrichment in early-stage knee OA, and that it consequently may represent a study group suitable for future pharmacological disease-modifying intervention. We assessed our patients at an interval of 2 years; this period of time being suggested as a minimum for clinical trials of disease modification in OA to detect both structural and symptom change [29].

It could be that the findings reported here are valid only for post-injury, secondary OA, or for this particular cohort. However, the criteria and delimitations for posttraumatic OA compared to primary OA have recently been shown to be much less clear than thought [13,14,30], and meniscal pathology is common also in primary, garden-variety, knee OA [31]. Tibiofemoral OA was observed in 53 out of 133 patients who were underwent radiographic examination. Isolated patellofemoral OA was rare and, since it did not affect the final results, was not taken into account. A further argument favoring the general applicability of the present results is the concordance of our findings with other longitudinal studies on OA [2-5,32].

### Methodological issues

We applied the criteria for minimal perceptible clinical improvement (MPCI) obtained for the WOMAC; since KOOS contains the WOMAC items and is similar in format. The KOOS subscale ADL is equivalent to the WOMAC subscale Function, while new items have been added to the KOOS subscales Pain and Symptoms. The dimensions assessed by the KOOS subscales Sport and Recreation Function and knee-related Quality of Life are not assessed by the WOMAC. The MPCI for the WOMAC is in the range of 8 to 12 points on a 0–100 scale [20].

This threshold coincides with the change observed in KOOS scores between 3 and 6 months postoperatively when assessing rehabilitation following reconstruction of the anterior cruciate ligament and concurs with the OARSI definition of moderate improvement in the knee pain assessment for clinical trials in OA [18,22]. However, the OARSI responder criteria were designed for the evaluation of the patient's response to oral NSAID and intra-articular treatment and may differ for other interventions.

It may be argued that the subject-related changes observed in this study represent inherent instrument instability. However, validation studies of KOOS support the reproducibility and stability of the KOOS instrument [17-19]. Test-retest data on the KOOS subscale pain obtained from 75 patients about to undergo knee arthroscopy [17] was used to determine the number of subjects improving, deteriorating or not changing over an average period of 5 days.

The proportion of subjects changing over 5 days was approximately half of that changing over 2 years in the present study, in further support that the variation in the present study cannot be explained solely by instrument noise (data not shown). A 'frame shift' in the priorities of the individual patient may occur during long term studies. However, we suggest that a significant frame shift is unlikely to have occurred over this 2 year study period of a cohort with a mean age of 50 years.

Significant change of KOOS scores over time were noted in 1/3 of the cohort studied. About half of those who changed clinically improved. This was true in particular for patients with lower (worse) baseline scores. It is thus possible that the lower proportion of 'changers' among those with better baseline scores may have been, at least in part, due to a ceiling effect.

## Conclusions

We conclude that despite unchanged group mean scores over 2 years, pain, function and quality of life change considerably over time in individuals, in this study cohort enriched in incipient and early-stage knee OA. These findings may be applicable also to other at risk patient groups in different phases of OA development, and motivate further careful examination of the natural history of OA, as well as properties of the OA outcome instrument used. We suggest that longitudinal OA study data should be analyzed both on the individual and group level.

Our findings may have further relevance to clinical trials of OA that seek to document long term benefit in the form of symptom improvement and structural improvement. It is clear that much additional effort will need to be spent on selection of groups at high risk of progression of symptoms and structural joint change, and the identification of predictors for deterioration. Our results also suggest that the use of responder criteria may be an important aspect of analyzing the outcome of such trials [22,33].

## Authors' contributions

EMR and LSL planned study and collected the data. PTP performed the statistical analysis and drafted the manuscript. ME formed the database of patients and participated in the statistical analysis. ME, EMR, LSL corrected the manuscript. All authors read and approved the manuscript
